# Heme oxygenase, biliverdin reductase, and bilirubin pathways regulate oxidative stress and insulin resistance: a focus on diabetes and therapeutics

**DOI:** 10.1042/CS20242825

**Published:** 2025-01-28

**Authors:** Wang-Hsin Lee, Zachary A. Kipp, Sally N. Pauss, Genesee J. Martinez, Evelyn A. Bates, Olufunto O. Badmus, David E. Stec, Terry D. Hinds

**Affiliations:** 1 Drug & Disease Discovery D3 Research Center, Department of Pharmacology and Nutritional Sciences, University of Kentucky College of Medicine, Lexington, KY, USA; 2 Department of Physiology & Biophysics, Cardiorenal and Metabolic Diseases Research Center, University of Mississippi Medical Center, Jackson, USA; 3 Barnstable Brown Diabetes Center, University of Kentucky College of Medicine, Lexington, KY, USA; 4 Markey Cancer Center, University of Kentucky, Lexington, KY, USA

**Keywords:** Bilir, Blvra, Hmox1, Inflammation, Masld, Metabolism, Obesity, Reactive oxygen species, Ugt1a1, Urobilin

## Abstract

Metabolic and insulin-resistant diseases, such as type 2 diabetes mellitus (T2DM), have become major health issues worldwide. The prevalence of insulin resistance in the general population ranges from 15.5% to 44.6%. Shockingly, the global T2DM population is anticipated to double by 2050 compared with 2021. Prior studies indicate that oxidative stress and inflammation are instrumental in causing insulin resistance and instigating metabolic diseases. Numerous methods and drugs have been designed to combat insulin resistance, including metformin, thiazolidinediones (TZDs), sodium-glucose cotransporter 2 inhibitors (SGLT2i), glucagon-like peptide 1 receptor agonists (GLP1RA), and dipeptidyl peptidase 4 inhibitors (DPP4i). Bilirubin is an antioxidant with fat-burning actions by binding to the PPARα nuclear receptor transcription factor, improving insulin sensitivity, reducing inflammation, and reversing metabolic dysfunction. Potential treatment with antioxidants like bilirubin and increasing the enzyme that produces it, heme oxygenase (HMOX), has also gained attention. This review discusses the relationships between bilirubin, HMOX, and insulin sensitivity, how T2DM medications affect HMOX levels and activity, and potentially using bilirubin nanoparticles to treat insulin resistance. We explore the sex differences between these treatments in the HMOX system and how bilirubin levels are affected. We discuss the emerging concept that bilirubin bioconversion to urobilin may have a role in metabolic diseases. This comprehensive review summarizes our understanding of bilirubin functioning as a hormone, discusses the HMOX isoforms and their beneficial mechanisms, analyzes the sex differences that might cause a dichotomy in responses, and examines the potential use of HMOX and bilirubin nanoparticle therapies in treating metabolic diseases.

## Introduction

Over the past few decades, insulin resistance has become a major health issue worldwide [3]. A report by the American Diabetes Association indicated that ~70% of individuals with insulin resistance eventually develop type 2 diabetes mellitus (T2DM) [1]. If left untreated, patients with T2DM have a 16% increased risk of all-cause mortality and an 18% increased risk of cardiovascular death [2,3].

The global T2DM population is predicted to at least double by 2050 compared with 2021 [4]. Thus, the development of insulin-sensitizing strategies is still in high demand. To this end, numerous strategies and drugs have been developed, including metformin, thiazolidinediones (TZDs), sodium-glucose cotransporter 2 inhibitors (SGLT2i), glucagon-like peptide 1 receptor agonists (GLP1RA), and dipeptidyl peptidase 4 inhibitors (DPP4i). Regulating factors in the insulin signaling pathway is a major therapeutic strategy for improving insulin sensitivity. However, it is not the only way to reverse insulin resistance. Signaling dysfunction that stimulates the development of peripheral insulin resistance can commence in the liver [5]. Hepatocytes in the liver are primarily responsible (~90%) for removing insulin from the blood, which is secreted from the pancreas [5]. Insulin-resistant hepatocytes also threaten whole-body health as they can increase fat accumulation, reduce insulin clearance, increase circulating insulin half-life [6], and stimulate gluconeogenesis [6]. All of these lead to metabolic dysfunction-associated steatotic liver disease (MASLD) [5,7-13], hyperinsulinemia, and hyperglycemia. MASLD-derived diseases are life-threatening issues that are receiving more attention [9,10], as MASLD-related mortality has increased 8.5 times in the U.S. over the past 23 years [14]. If left untreated, MASLD may progress to metabolic dysfunction-associated steatohepatitis (MASH) with liver fibrosis that can manifest into cirrhosis [15,16], impacting the health and shortening the life of the patient.

Therapies also target other pathways that improve insulin signaling, such as inhibiting oxidative stress, a known culprit in developing insulin resistance [17]. Excessive reactive oxygen species (ROS) production has been shown to perturb insulin signaling pathways and inhibit cellular stimulation of insulin [17]. Individuals with obesity and overnutrition have elevated circulating glucose and free fatty acids (FFAs), which raise intracellular ROS production [18]. Thus, stopping the negative impact of oxidative stress on the insulin signaling pathway helps reverse insulin resistance and metabolic dysfunction [19,20]. Elevated plasma bilirubin concentrations were associated with favorable outcomes in metabolic health and oxidative stress biomarkers [21].

HMOX is an enzyme that controls heme degradation and the production of biliverdin, carbon monoxide (CO), and ferritin. Biliverdin is rapidly reduced to bilirubin [22], a strong antioxidant and fat-burning hormone [23]. The HMOX generation of bilirubin suppresses inflammation and improves insulin sensitivity in individuals with obesity or diabetes experiencing oxidative stress [24-29]. Thus, the HMOX production of bilirubin benefits insulin sensitivity and metabolism. Some published work has demonstrated that the glucuronyl UGT1A1 enzyme that conjugates bilirubin is elevated in obesity [23], causing a reduced bilirubin plasma half-life and lowering circulating levels. People with obesity typically have low plasma bilirubin (hypobilirubinemia) [23], which might be due to exaggerated liver turnover, causing more bilirubin conjugation and excretion into the intestines via the biliary system [30,31]. Within the intestines, bilirubin is catabolized into metabolites urobilin and stercobilin [31]. This accelerated process may contribute to the raised plasma urobilin levels observed in women and men with insulin resistance [32]. Kipp *et al.* found that plasma urobilin levels positively correlated with insulin resistance, with higher urobilin equating to higher HOMA-IR, a measure of insulin resistance.

This review discusses insulin resistance (1) mechanisms, (2) the actions and side effects of existing treatments, (3) the potential of HMOX and bilirubin as therapeutic targets against it, and (4) we discuss, when possible, the sex-specific effects of their signaling.

## Insulin resistance, oxidative stress, and bilirubin

### Insulin forms and signaling mechanisms

**Figure 1:**
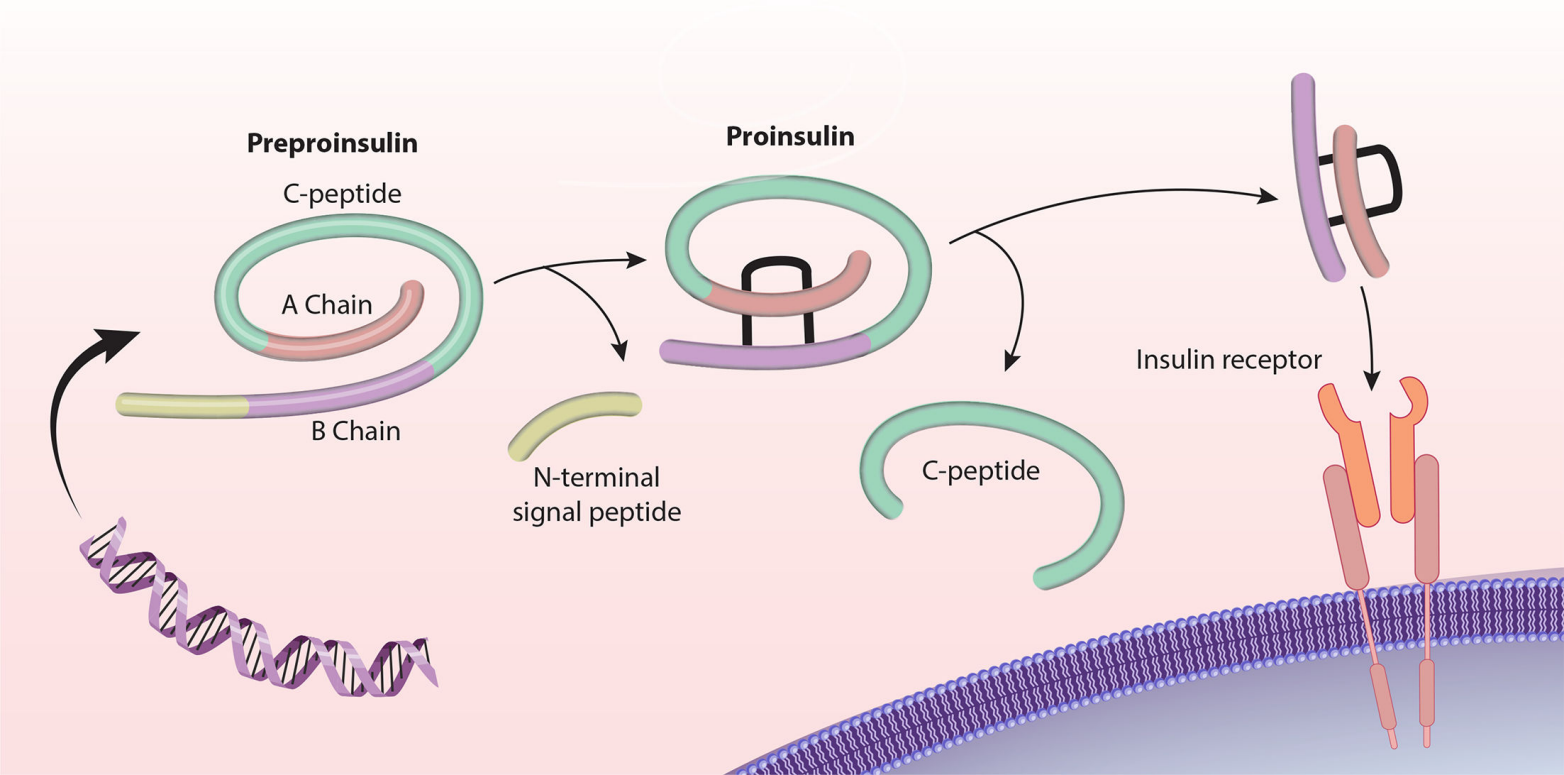
The maturation of pre-insulin into a signaling molecule. Insulin is initially synthesized as pre-proinsulin. After removing the N-terminal signal peptide, disulfide bonds form within the molecule, forming proinsulin. Mature insulin is produced when the C-peptide is cleaved from proinsulin. At this stage, insulin is capable of binding to the insulin receptor.

Insulin is initially produced as pre-proinsulin containing three structural domains: A, B, and C peptide domains. It is processed as proinsulin in pancreatic cells (Figure 1) [5]. The C-peptide on the proinsulin molecule is cleaved and released, forming mature insulin with only the A and B domains in the bloodstream [33]. This version of insulin binds to activate the insulin receptor. The ratio of insulin to C-peptide levels can indicate whether there is a pancreatic or liver issue affecting insulin signaling [34]. Insulin resistance occurs when blood glucose uptake fails to respond to insulin signaling, resulting in reduced insulin responsiveness compared with normal stimulation [35].

### Insulin-regulated kinase pathways

**Figure 2:**
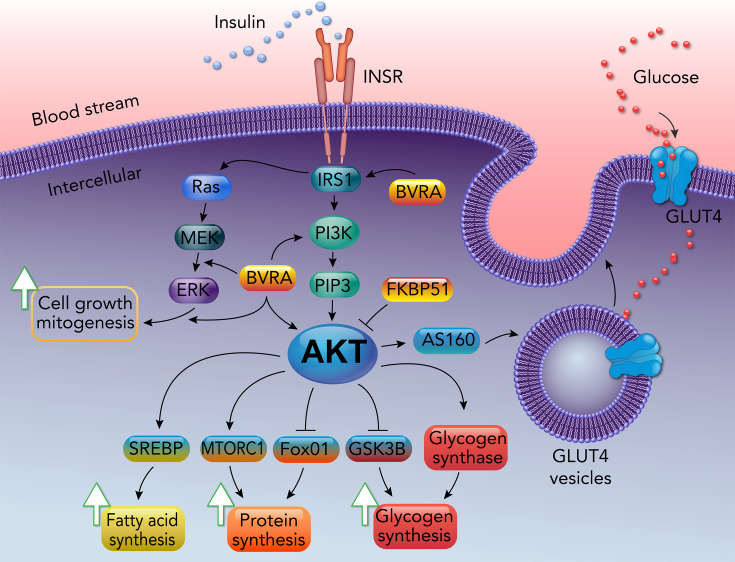
The canonical insulin signaling pathway. Insulin regulates the metabolism of glucose, fatty acids, proteins, and glucose uptake through the PI3K/AKT pathway. The insulin signaling’s Ras/MEK/ERK wing regulates cell proliferation and mitogenesis.

The insulin receptor is a transmembrane tyrosine kinase located in the plasma membrane. Factors that influence insulin sensitivity or resistance might target the insulin signaling pathway, including insulin receptor substrate (IRS), phosphatidylinositol 3-kinase (PI3K), protein kinase B (AKT), and glucose transporter 4 (GLUT4) (Figure 2) [5,36]. Proteins, such as AKT substrate of the 160 kDa (AS160), FK506-binding protein-51 (FKBP51), and BVRA, have been shown to regulate insulin sensitivity [5]. While BVRA and AS160 influence insulin sensitivity, FKBP51 drives insulin resistance by binding to AKT with PHLPP, thereby reducing phosphorylation levels [37-40].

Canonical insulin signaling compromises two major pathways: the PI3K/AKT and the mitogen-activated protein kinases (MAPK)/extracellular signal-regulated kinases (ERK) signaling cascades (reviewed in more detail in [5]). The PI3K/AKT pathway controls the metabolism of sugars, proteins, and lipids, whereas the ERK signaling mostly regulates cell growth [41]. When insulin binds to its receptor, the insulin receptor tyrosine kinase phosphorylates IRS, activating the PI3K/AKT pathway by phosphorylation. The increase in phospho-AKT (pAKT) activates glycogen synthase (GYS), the mammalian target of rapamycin complex 1 (mTORC1), and sterol regulatory element-binding protein 1 (SREBP-1C) and inhibits glycogen synthase kinase‐3 (GSK3) and forkhead box protein O1 (FOXO1) [42], which results in increased glycogen, protein, and lipid synthesis [5]. pAKT also activates AS160 signaling, increasing GLUT4 vesicle fusion with the plasma membrane and increasing glucose uptake in muscle cells and adipocytes [36,43]. Regulation of these pathways may directly affect glucose uptake.

### Reactive oxygen species and insulin resistance

**Figure 3:**
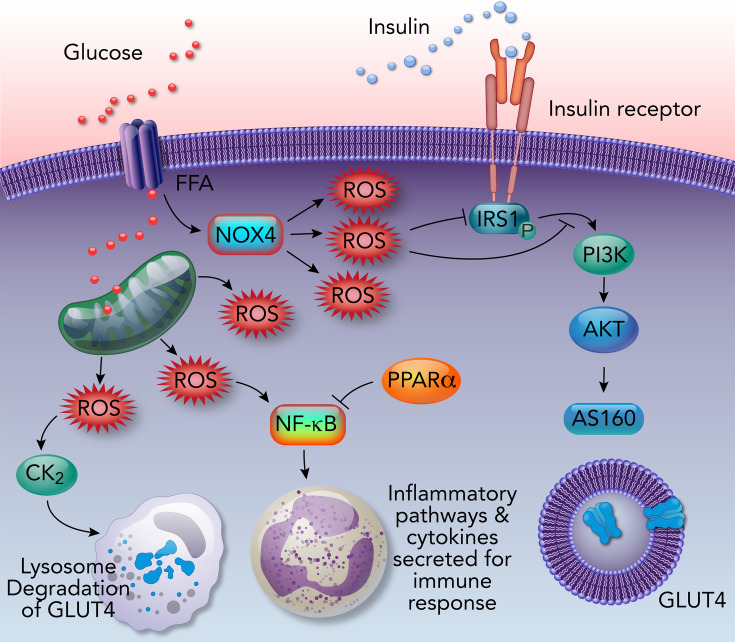
Oxidative stress impairs insulin signaling. The diagram shows how oxidative stress interrupts insulin signaling and reduces insulin sensitivity. Excessive glucose and FFA increase endogenous ROS through NOX4 and mitochondria. These ROS can inhibit insulin signaling by blocking the activation of IRS and the recruitment of PI3K. ROS can also activate CK2 to facilitate GLUT4 degradation in lysosomes.

Oxidative stress inhibits insulin signaling and sensitivity [17,44]. In addition, ROS, such as hydrogen peroxide (H_2_O_2_), have been shown to inhibit IRS function and the recruitment of PI3K, which blocks the activation of insulin signaling (Figure 3) [45]. Excessive lipids from obesity inflict oxidative damage by enhancing FFA oxidation in the mitochondria, raising ROS levels [46,47]. Increased ROS also causes mitochondrial fission, contributing to insulin resistance in mouse skeletal muscle [48]. ROS can directly or indirectly interact with the insulin signaling pathway and induce insulin resistance. Increasing ROS triggers phosphorylation of the transcription factor, NF-κB (nuclear factor-κB) [49], activating inflammatory pathways [46]. Stimulating NF-κB transcriptional activity also increases SREBP-1 expression, leading to *de novo* lipid production through increased fatty acid synthase (FASN) expression [50,51]. PPARα (peroxisome proliferator-activated receptor alpha) is an NF-κB/SREBP-1/FASN axis inhibitor and stimulates fat-burning β-oxidation, inhibiting lipogenesis, promoting ketogenesis, and enhancing fat utilization [52-55]. Activation of PPARα increases HMOX1 expression [56] and lowers ROS levels [57,58].

Major sources of oxidative stress in metabolic dysfunction are derived from excessive plasma glucose and FFAs [59], which drive mitochondria production of ROS [18]. Increased ROS production from NADPH oxidase (NOX) has been shown to cause insulin resistance [60]. Increased intracellular ROS levels activate casein kinase-2 (CK2), resulting in GLUT4 degradation in lysosomes instead of being transported to the plasma membrane, which reduces glucose uptake (Figure 2) [61]. The decrease in glucose uptake and cellular responses to insulin is a known characteristic of insulin resistance.

### Bilirubin as an antioxidant

Bilirubin is one of the most potent endogenous antioxidants in the body. Early studies by Stocker described that bilirubin’s unique chemical structure of an extended system of conjugated double bonds and a reactive hydrogen atom possesses remarkable antioxidant capacity [62]. Additional studies also demonstrated that free bilirubin and albumin-bound bilirubin are physiological antioxidants [63]. Bilirubin can directly scavenge ROS and inhibit NADPH oxidase, the major enzyme responsible for producing superoxide anions [64]. Mice deficient for biliverdin reductase-A (BVRA), the rate-limiting enzyme responsible for the reduction of biliverdin and the generation of bilirubin, are characterized by high plasma markers of ROS production [65]. In addition, patients with Gilbert’s polymorphism, resulting in increased serum levels of bilirubin, exhibit increased serum antioxidant status and are resistant to serum oxidation, which may offer protection against cardiovascular disease [66]. Bilirubin is not only an important antioxidant on its own, but it also interacts with vitamin E to increase antioxidant capacity. Bilirubin aids in regenerating membrane-bound vitamin E to increase its antioxidant capacity [67]. The results of these studies demonstrate that bilirubin acts at multiple levels to serve as an important endogenous antioxidant [62].

## Heme oxygenase and biliverdin reductase affect insulin sensitivity

### Heme oxygenase and biliverdin reductase gene regulation and protein functions

**Figure 4:**
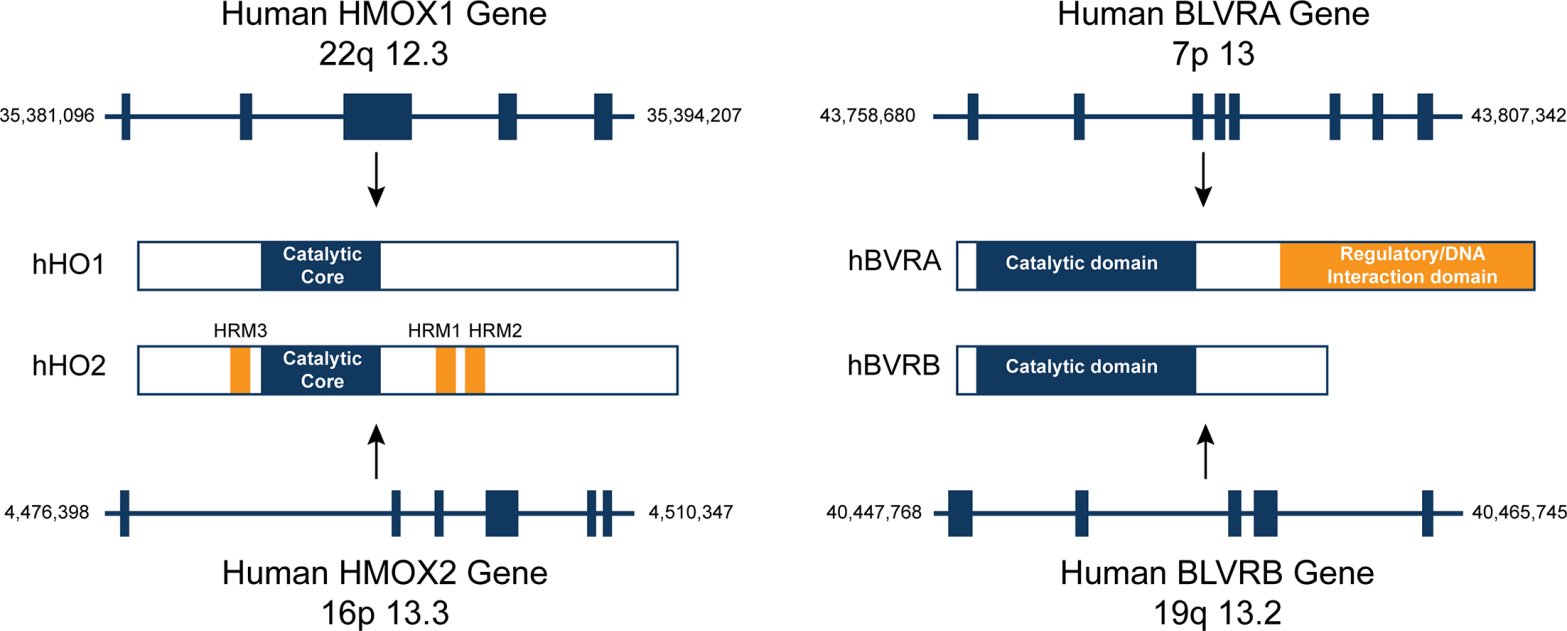
The gene and protein domain structures of human HMOX1, HMOX2, BLVRA, and BLVRB. Human HMOX1 and HMOX2 are encoded on chromosomes 22 and 16, respectively, with HMOX1 comprising five exons and HMOX2 comprising six exons. Their domain structures are similar but not identical, primarily due to the presence of three heme regulatory motifs (HRMs) found exclusively in HMOX2. Human BLVRA and BLVRB are encoded on chromosomes 7 and 19, respectively. BLVRA consists of eight exons, while BLVRB contains five exons. A difference in their peptide domain structure is in the regulatory/DNA interaction domain, which is found only in BVRA.

Heme oxygenases are enzymes encoded by the *HMOX1* and *HMOX2* genes (Figure 4). HMOX1 is the inducible isoform that can be widely expressed in organs and tissues, including insulin-sensitive organs like the liver, muscles, and adipose. On the other hand, HMOX2 is expressed at the highest in the brain [68] and testis [69]. The major function of HMOX is to catalyze heme degradation to produce CO, ferritin, and biliverdin during the catabolism process [70]. Since HMOX prevents heme accumulation and toxicity [71,72] and has anti-inflammatory effects [73], it is a well-accepted cytoprotective protein. HMOX1 expression is inducible by many cellular events, including stress [74].

The *HMOX1* promoter contains DNA regions called response elements that regulate the activation or suppression of the gene. The antioxidant response element (ARE) in the *HMOX1* promoter is suppressed when the BACH1 (BTB domain and CNC homolog 1) protein is bound, which occurs when the cell is not experiencing stress [75]. NRF2 (nuclear factor erythroid 2-related factor 2) replaces BACH1 in its binding site on the *HMOX1* promoter when stimulation exists, including proinflammatory signaling from NF-κB, interleukin 10 (IL-10), prostaglandins, and metabolic signaling like AMPK and hypoxia-inducible factor 1α (HIF-1α) [75], all leading to increased *HMOX1* gene expression [75]. Other stimulating factors include cell differentiation, which is associated with *HMOX1* expression. For example, *HMOX1* mRNA expression gradually decreases over the differentiation process of human adipocytes [76], which impacts adipogenesis [77].

**Figure 5:**
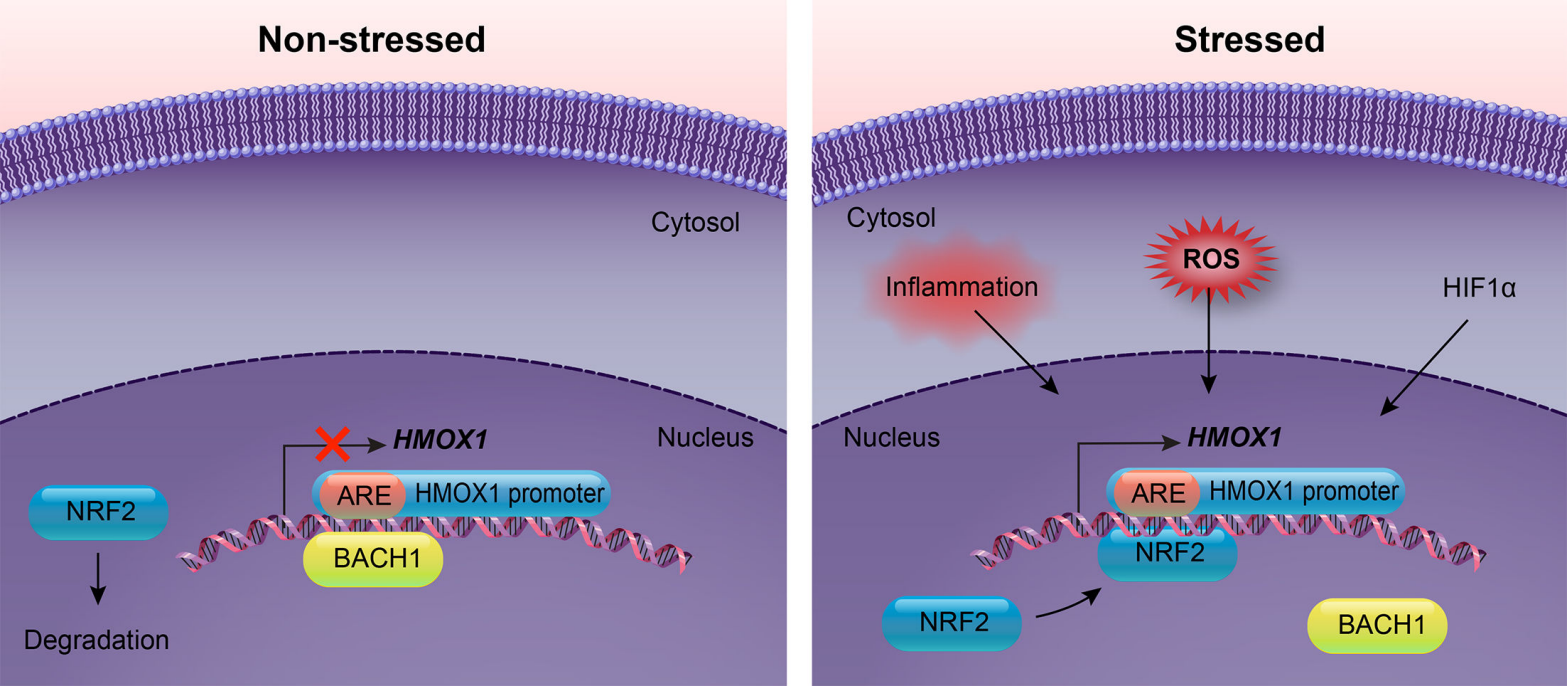
The regulation of the HMOX1 promoter. Under non-stressed conditions, the ARE binding site in the HMOX1 promoter is occupied by BACH1, which inhibits HMOX1 expression. During this time, NRF2 remains in the cytoplasm and undergoes degradation. Upon receiving stress signals such as HIF1α, ROS, and inflammatory cytokines, NRF2 is activated and translocates to the nucleus. In the nucleus, NRF2 replaces BACH1 on the ARE of the HMOX1 promoter, thereby inducing HMOX1 expression.

Hemin and cobalt protoporphyrin (CoPP) are well-known inducers of HMOX activity [78-82] and have been used in studies to increase *HMOX1* gene expression and enzyme activity (Figure 5) [82-85]. For inhibiting HMOX1 enzyme function, metalloporphyrins (MPs) and imidazole-dioxolane derivatives can be used as non-selective and selective inhibitors for HMOX1, respectively [86]. These findings have provided strategies for regulating HMOX1 functions, which improve the feasibility of using it as a therapeutic target. On the other hand, HMOX2 expression and function have long been considered constitutive [74] because scientists found it difficult to regulate HMOX2 [87]. However, it has been shown that HMOX2 expression is up-regulated by adrenal glucocorticoids and the activation of glucocorticoid response element (GRE) [88] in the *Hmox2* promoter [89]. Other than glucocorticoids, studies have shown that hypoxemia, opioids [87], and increased astrocytic Ca^2+^ [90] also regulate HMOX2 expression. In an in-depth report by Intagliata et al., compounds including MPs, azalanstat, ketoconazole, and terconazole have been shown to inhibit HMOX2 enzyme activities [91]. The same study also reported that menadione, known as vitamin K3, is a selective HMOX2 activator, and this was supported by Vukomanovic et al. [92]. Based on these findings, HMOX2 expression and function are no longer considered unregulatable and constitutive, indicating its potential as a novel treatment target.

**Figure 6:**
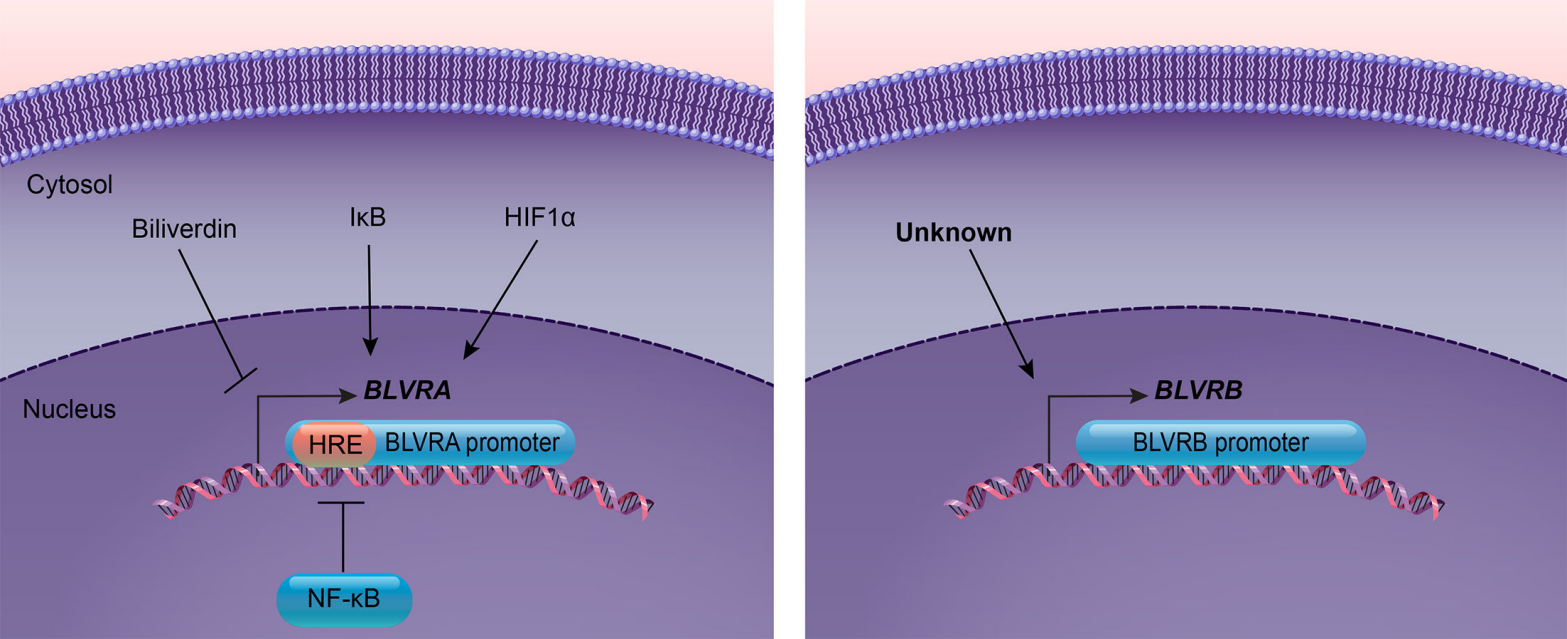
The regulation of BVR promoters. Factors such as biliverdin, IκB, and HIF1a regulate BLVRA expression (left panel). Transcription factors like NF-κB regulate BLVRA expression by its HRE binding site. Very little is known about what regulates BLVRB expression (right panel).

Biliverdin reductase (BVR) is a crucial enzyme in the heme degradation pathway, with its two isoforms encoded by genes *BLVRA* and *BLVRB* (Figure 6). The expression of *BLVRA* is positively regulated by hypoxia and IκB (inhibitor of nuclear factor kappa B) overexpression. In contrast, it is negatively regulated by Tumor Necrosis Factor α (TNFα)-induced NF-κB activation and biliverdin [93]. The *BLVRA* promoter contains four hypoxia-responsive elements (HREs), one of which binds to hypoxia-inducible factor-1α (HIF-1α), causing an increase in *BLVRA* expression under hypoxic conditions [93]. Hypoxia up-regulates *HMOX1* and *BLVRA* promoter activities via increased HIF-1α, suggesting a potential correlation between these critical enzymes in the heme degradation pathway. In contrast, the regulatory mechanisms governing the *BLVRB* promoter remain unknown. Further research is needed to elucidate the regulatory mechanisms of the *BLVRB* gene.

### Insulin signaling and the roles of heme oxygenase and biliverdin reductase

A major mechanism by which HMOX increases insulin sensitivity is by reducing oxidative stress. HMOX1-mediated CO production down-regulates NOX4 activity [94], suggesting it inhibits intracellular ROS production via NOX4. HMOX1 also produces biliverdin during heme degradation. Biliverdin and bilirubin are strong antioxidants that contribute to ROS scavenging and protect against the accumulation of intracellular oxidative stress [95]. Based on these functions, HMOX1 ameliorates ROS-induced interruption to insulin signaling by lowering NOX4 activity and neutralizing ROS via biliverdin and bilirubin. HMOX1 can also increase insulin sensitivity by regulating inflammation. Inhibiting HMOX1 activity causes increased serum proinflammatory cytokine levels in obese mice [82]. These cytokines include TNFα and interleukin-6 (IL-6), which have been considered to promote insulin resistance by inhibiting IRS activation by insulin receptor tyrosine kinase [13,34,96-99]. The same study found that HMOX1 induction by CoPP increased insulin sensitivity and glucose tolerance and decreased blood insulin levels.

Overall, HMOX1 increases insulin sensitivity by significantly lowering oxidative stress and inflammation. The other isoform, HMOX2, may play a similar role to HMOX1. It has been shown that HMOX2-null mice are obese and insulin-resistant [100]. However, the role of HMOX2 in regulating insulin sensitivity still needs to be fully understood, and more studies are necessary to determine how it affects insulin sensitivity.

### Activating heme oxygenase as a treatment for insulin resistance

The induction of *HMOX1* expression has been considered a potential treatment for reversing insulin resistance [72,101]. However, there is controversy about HMOX1’s role. A 2014 study by Jais et al. indicated that HMOX1 does not improve insulin sensitivity [101], while other studies showed that it helps promote insulin sensitivity. Hinds et al. [71] discussed this conundrum in an editorial, specifying why there may be differences between the findings in the animal studies. The HMOX1 knockout mice still expressed a small, truncated HMOX1 protein, which likely explains their variable results. The study by Jais et al showed that hepatocyte-specific HMOX1 knockout mice fed a high-fat diet (HFD) presented reduced insulin resistance and higher insulin receptor and AKT phosphorylation levels in the liver, muscle, and adipose tissues when injected with insulin compared with the wild-type mice [101]. The same study also showed that when they specifically knocked out HMOX1 in macrophages in mice, they were resistant to HFD-induced weight gain, glucose intolerance, insulin resistance, hepatic steatosis, and inflammation. Liao et al*.* showed that gluconeogenesis increases when overexpressing HMOX1 in hepatocytes and mice [102]. They also found that inhibiting HMOX1 reduces abnormal gluconeogenesis in IRS-1 and IRS-2 double-knockout mice. This single finding indicates that reducing HMOX1 levels may help reduce insulin resistance. However, more studies support the protective effects of HMOX1 against insulin resistance. For instance, it has been shown that losing *Hmox1* expression in mice increases insulin resistance [103]. In addition, increased HMOX1 expression protects rats from hyperglycemia and insulin resistance [28,104]. Wallner et al. reported the protective effects of HMOX1 by demonstrating a positive correlation between its catabolites and reduced body mass index (BMI) and inflammation in human subjects [105]. Wegiel et al. suggested that HMOX1 mitigates the metabolic syndrome through its antioxidant properties [73]. Additionally, circulating HMOX levels are associated with reduced inflammation and BMI in individuals with Gilbert’s syndrome, characterized by mild hyperbilirubinemia [105]. These findings indicate that HMOX1 protects against insulin resistance and related risk factors.

HMOX1’s protective effect is also supported by Burgess et al., who found that increasing HMOX1 function by treating mice with CoPP decelerated weight gain in obese male mice and decreased plasma glucose and proinflammatory cytokines in obese female mice [106]. They also found that CoPP-induction of HMOX1 increased pAKT and PPARγ levels in adipocytes from obese animals, indicating increased insulin sensitivity in adipocytes. Increased pAKT can also be found in bone marrow-derived mesenchymal stem cells (BMSCs) with lentivirus-induced HMOX1 overexpression [107]. Another study showed that HMOX1 induction by hemin increased the lower pAKT levels in the aortas in diabetic mice, which is considered to occur through increased bilirubin levels [108]. Thus, although some studies show that HMOX1 does not contribute to insulin sensitivity, published work still supports HMOX1’s potential to be a T2DM treatment target. One aspect to consider further is that bilirubin may be the primary contributor to HMOX1’s antidiabetic effects. In the next section, we will explore the role of bilirubin in insulin sensitivity.

## Bilirubin and insulin sensitivity

### Bilirubin generation and its cellular function

**Figure 7:**
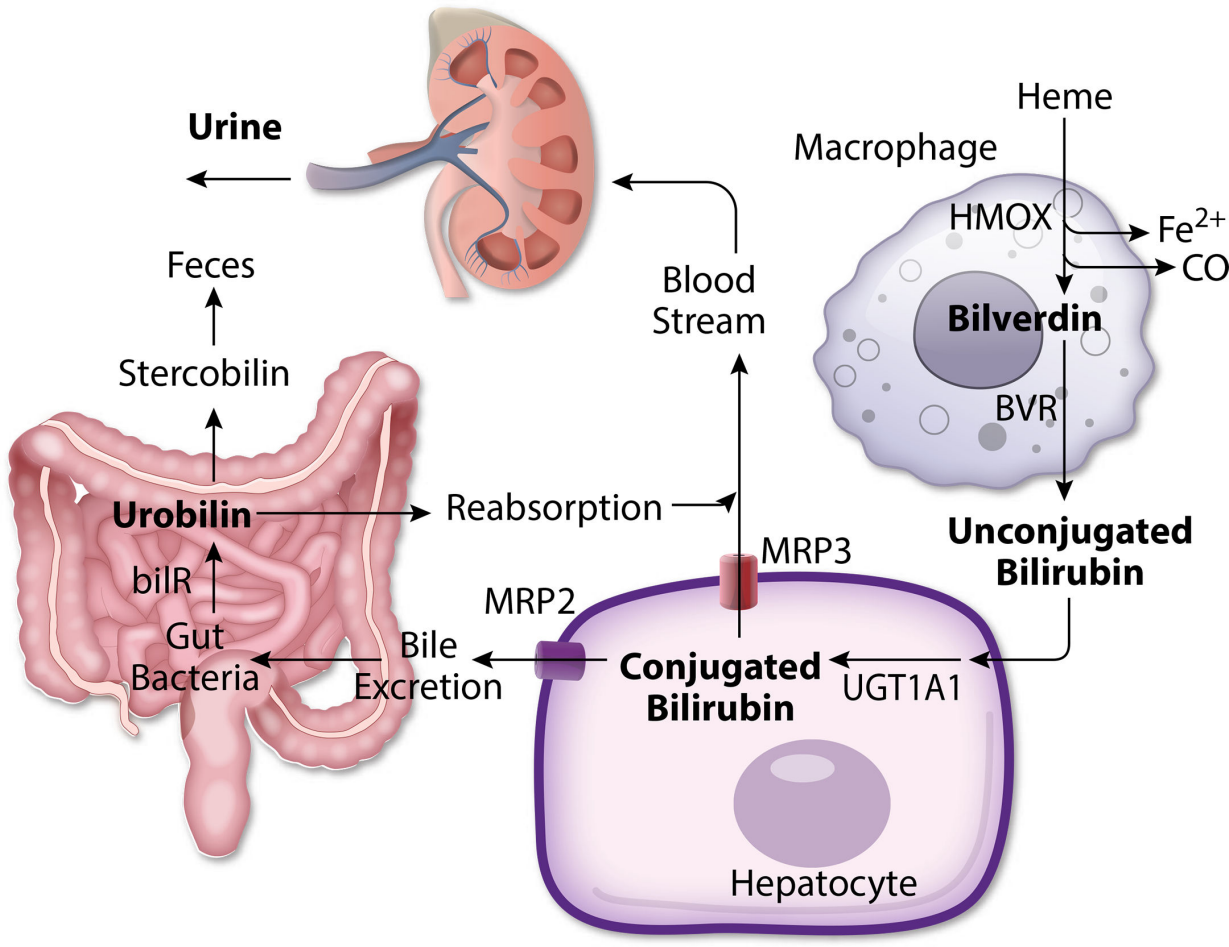
The schematic overview of the heme degradation pathway. The overview of heme degradation and bilirubin metabolism. Heme is converted into bilirubin via HMOX and biliverdin reductase in macrophages and transported to hepatocytes. Hepatocytes conjugate bilirubin using UGT1A1 and excrete it through either MRP2 to the gut or MRP3 back to the bloodstream. The bacterial metabolite of conjugated bilirubin, urobilin, can be reabsorbed back into the body or excreted via feces. The reabsorbed urobilin and the conjugated bilirubin in the bloodstream can be excreted through urine.

Bilirubin is an endogenous metabolite derived from the degradation of heme. During the breakdown of erythrocytes and heme degradation in the spleen, bone marrow, and hepatic Kupffer cells, heme is first converted to biliverdin by HMOX and then to bilirubin by BVR [70,109] (Figure 7). The unconjugated bilirubin form (UCB) is very hydrophobic and is transported through systemic circulation bound to albumin. The liver is the next stop for UCB and the major organ responsible for bilirubin excretion. In the hepatocyte, UCB will be converted to glucuronide-conjugated bilirubin by the UDP-glucuronyltransferase (UDP-GT) encoded by the *UGT1A1* gene [110] (Figure 7). Bilirubin has many isoforms, and bilirubin IXα is the major form in adult mammals [109]. The variable bilirubin isoform formation is determined by BVR, where BVR isoform A (BVRA) produces bilirubin IXα and BVR isoform B (BVRB) produces bilirubin IXβ [111]. Bilirubin IXα needs conjugation through the UGT1A1 enzyme to be excreted, while bilirubin IXβ is more water-soluble and can be excreted in bile without conjugation [111]. After excretion into the gut through bile, bilirubin can be converted to other metabolites, such as urobilinogen and urobilin, by gut bacteria. The bilirubin reductase (bilR) protein, which functions as the enzyme that catabolizes bilirubin to urobilin, was recently described by Hall and colleagues [112]. While they identified numerous species that generate urobilinogen, the specific bacteria that produce the different urobilinogen isoforms is unknown. More investigations in this area are essential to understanding urobilinogen’s and urobilin’s functions. Urobilin can be reabsorbed in the gut [30], and some will be excreted through feces and converted to stercobilin, which gives feces its color (Figure 7) [31].

It has long been viewed that bilirubin is harmful, as it was considered a marker of liver damage for centuries [113]. However, recent studies suggest that urobilin may be the culprit. Normal plasma total bilirubin levels usually range from 0.2 to 1.2 mg/dL (3.42–20.52 µM) [23]. Health issues, such as pathological jaundice, can arise when plasma bilirubin levels exceed 150 µM [23]. However, mild hyperbilirubinemia, where bilirubin levels are 18–58 µM, has protective actions against many diseases [23,31,113-115]. A deficiency in hepatic bilirubin production from BVR induces MASLD and inflammation, as demonstrated by separate research groups, one using liver-specific BVRA knockout mice [13] and the other using global BVRA knockout mice [65]. Both groups concluded that hepatic PPARα experienced hyperphosphorylation of the inhibitory serine 73, reducing its activity. An adipose-specific knockout of BVRA in mice induced insulin resistance, inflammation, and reduced mitochondrial function in iWAT but not in brown fat tissue [116]. Mild hyperbilirubinemia drives fatty acid oxidation and reduces adiposity [23,55,117,118].

### The bilirubin receptor

**Figure 8:**
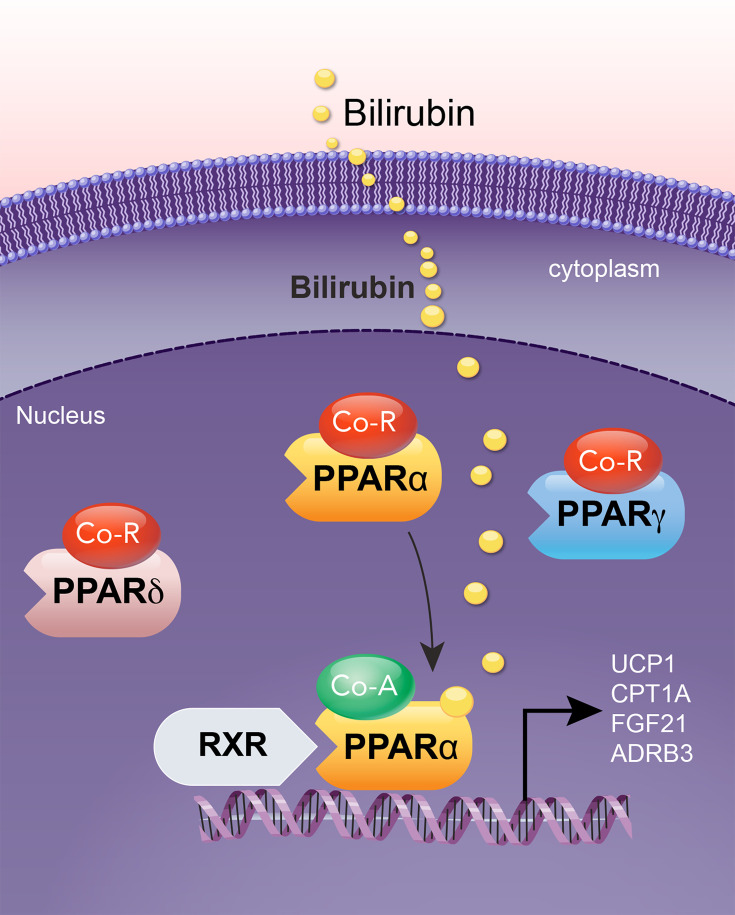
Bilirubin selectively binds PPARα as its physiological receptor. Bilirubin selectively binds to and activates PPARα within the PPAR family. Upon binding to bilirubin and activation, PPARα recruits co-activators and retinoid X receptor (RXR) to form a transcription-regulating complex. This complex binds to DNA at the peroxisome proliferator response element (PPRE), thereby promoting the expression of PPARα target genes, including uncoupling protein 1 (UCP1), carnitine palmitoyltransferase 1A (CPT1A), fibroblast growth factor 21 (FGF21), and adrenergic receptor beta 3 (ADRB3).

The primary receptor activated by bilirubin within its normal physiological ranges (10–58 µM) is PPARα (Figure 8), a nuclear receptor that promotes fat-burning and improves insulin sensitivity [23,119-123]. Bilirubin has been shown to bind directly to the ligand binding domain (LBD) of PPARα [119,123-125]. Bilirubin selectively activates PPARα and not the other PPAR isoforms PPARγ and PPARδ [123,124]. Competitive binding assays with bilirubin and PPARα agonist fenofibrate demonstrated that they bind the LBD, with fenofibrate displaced bilirubin’s binding [123,124]. Obese men and women with insulin resistance have reduced plasma bilirubin levels [32]. Bilirubin levels increase with exercise [126-128], which could be important for reducing fat accumulation. Bilirubin nanoparticle treatments significantly decreased adiposity and improved liver function in obese mice [55,118,124].

The PPARα activation by bilirubin contributes to improved insulin sensitivity, and many studies support this correlation. For example, one study showed that higher serum bilirubin levels are associated with reduced insulin resistance in individuals from China with obesity [129]. In addition, higher serum bilirubin levels have been reported to enhance insulin sensitivity in a Chinese-based study [130]. In the U.S. population, a study based on the National Health and Nutrition Examination Survey (NHANES) found that higher serum total bilirubin is associated with a lower risk of diabetes [131]. The benefits of higher bilirubin levels can also be found in another clinical study among 176 Japanese T2DM subjects, which showed that lower serum bilirubin levels are associated with higher diastolic blood pressure and visceral fat accumulation [132]. These findings all show that it is valuable to understand more about bilirubin’s regulation of insulin sensitivity.

### Hyperbilirubinemia protects against insulin resistance

Gilbert’s syndrome, a polymorphism in the *UGT1A1* promoter (UGT1A1*28) that reduces its expression by ~50% and affects nearly 10% of the human population, is the most common hereditary cause of hyperbilirubinemia [23,133,134]. People with Gilbert’s syndrome have plasma bilirubin levels that are 2–3-fold higher than the normal population but do not have any known side effects, such as liver diseases. People with Gilbert’s polymorphism (UGT1A1*28) exhibited significantly lower plasma proinflammatory cytokines, triglycerides, and cholesterol levels, and reduced obesity rates [135]. Studies in the humanized Gilbert’s syndrome mouse model, which has the UGT1A1*28 human gene locus and the mouse gene removed and exhibits mild hyperbilirubinemia, showed that when fed a high-fat diet, they had significantly less obesity, adiposity, and inflammation [117,136]. Mice with the humanized Gilbert’s polymorphism fed a high-fat diet did not develop glucose intolerance compared with control mice, who ate the same diet, as measured via glucose tolerance test (GTT) [117]. The independent groups that studied the humanized Gilbert’s syndrome mouse model found that they had significantly higher hepatic PPARα levels [117,136]. This was also found in people with the Gilbert’s syndrome polymorphism, who had increased PPARα and ATP-dependent AMP-activated protein kinase (AMPK), which they concluded were associated with leaner body weights and improved insulin sensitivity [137].

### Catabolism of bilirubin and development of insulin resistance

In 1997, Torgersen *et al*. first described that obese humans typically exhibit hypobilirubinemia and significantly raised aspartate aminotransferase (AST) and alanine aminotransferase (ALT) liver dysfunction biomarkers [138]. Obese rodents also experience hypobilirubinemia [124], which could be attributed to increased hepatic UGT1A1 [139,140], which conjugates bilirubin for excretion into the gut. In the intestine, the microbiome converts bilirubin to urobilinogen, which is rapidly oxidized to urobilin [30]. In humans, plasma urobilin is positively associated with insulin resistance [32], oxidized low-density lipoprotein (LDL) levels [141], T2DM [32,142,143], and all-cause mortality in diabetic patients [142]. Walker et al. observed that obese mice have significantly increased cecal urobilin content than lean controls [144]. Bates *et al*. suppressed UGT1A1 in obese mice via an N-acetylgalactosamine (GalNAc) labeled RNAi system and found that plasma bilirubin was considerably higher and urobilin was lower [145]. This resulted in significantly better insulin sensitivity and reduced plasma insulin levels than vehicle-treated obese mice [145]. These findings suggest that increasing plasma bilirubin and decreasing urobilin levels may benefit patients experiencing insulin resistance or T2DM. The role of urobilin in metabolic and cardiovascular diseases has been reviewed further (discussed in Kipp *et al*. [30]). More investigations are needed to better understand how urobilin may be involved in insulin resistance and metabolic dysfunction.

### Targeting bilirubin catabolism to increase its half-life as a treatment for insulin resistance

Lower bilirubin levels are associated with worse outcomes in metabolic dysfunction, such as obesity, insulin resistance, and T2DM [129,130]. Numerous studies have shown supportive findings by increasing bilirubin levels in animals. For instance, in diet-induced obese (DIO) mice, 14 days of bilirubin treatment via intraperitoneal injection improved the results of GTT and ITT [146]. These beneficial effects of bilirubin treatment were also observed in another investigation using *db/db* mice and DIO mice [147]. Additionally, this study found that a 14-day course of bilirubin injections increased pAKT levels in muscle tissue, reduced endoplasmic reticulum (ER) stress markers, and decreased macrophage infiltration in adipose tissue [147]. Bilirubin nanoparticle treatment has been shown to reduce diet-induced hepatic steatosis, suggesting its potential to alleviate hepatic insulin resistance [55,148], indicating the potential to mitigate hepatic insulin resistance. Other than treating with bilirubin and bilirubin nanoparticles, new treatment targets can also be developed to regulate bilirubin’s catabolism, especially the excretion pathway. Reducing bilirubin excretion in the gut to lower urobilin production and reabsorption may improve insulin sensitivity. It has been shown that inhibiting hepatic UGT1A1 increases plasma bilirubin levels and reduces plasma urobilin levels in mice [145]. In addition, they found that inhibiting hepatic UGT1A1 reduced liver fat content, blood glucose, and insulin levels. Increased urobilin levels have been associated with obesity and T2DM [32]. This finding supports targeting hepatic UGT1A1 as an antidiabetic treatment.

In humans, other than UGT1A1, targeting multidrug resistance proteins 2 (MRP2) and 3 (MRP3) could also be a potential strategy, but problems would likely arise as it could reduce bilirubin excretion into bile. There is an inherited disorder of reduced bilirubin excretion into bile called Dubin–Johnson syndrome (DJS), a benign disorder of bilirubin metabolism. MRP2 and MRP3 are transporters that excrete conjugated bilirubin into the bile canaliculus and extracellular fluid [149] (Figure 7). Inhibiting MRP2’s expression may increase bilirubin accumulation in hepatocytes and promote PPARα activation, which increases hepatic insulin sensitivity. However, inhibiting MRP2 and MRP3 will likely cause conjugated bilirubin to accumulate, and this form does not activate PPARα.

Studies have shown that excipients like Cremophor® EL, Cremophor® RH, Pluronic® F127, Maisine® 35–1, and β-cyclodextrin can significantly decrease the efflux of MRP2 substrate in Caco-2 cells [150,151]. In addition, Cremophor® EL enhances MRP3-mediated transportation [152]. The decrease in bilirubin excretion into the gut reduces urobilin production and decreases the clearance rate of bilirubin in urine (Figure 8). However, more studies are needed on the impact of suppressing UGT1A1, or MRP2 and MRP3, before determining its efficacy in reducing insulin resistance and how it might impair other factors it regulates, such as the conjugation of steroids [153] and other molecules.

## Treatments for insulin resistance and impact of heme oxygenase pathway

### Existing treatment strategies for insulin resistance in type II diabetes

**Figure 9:**
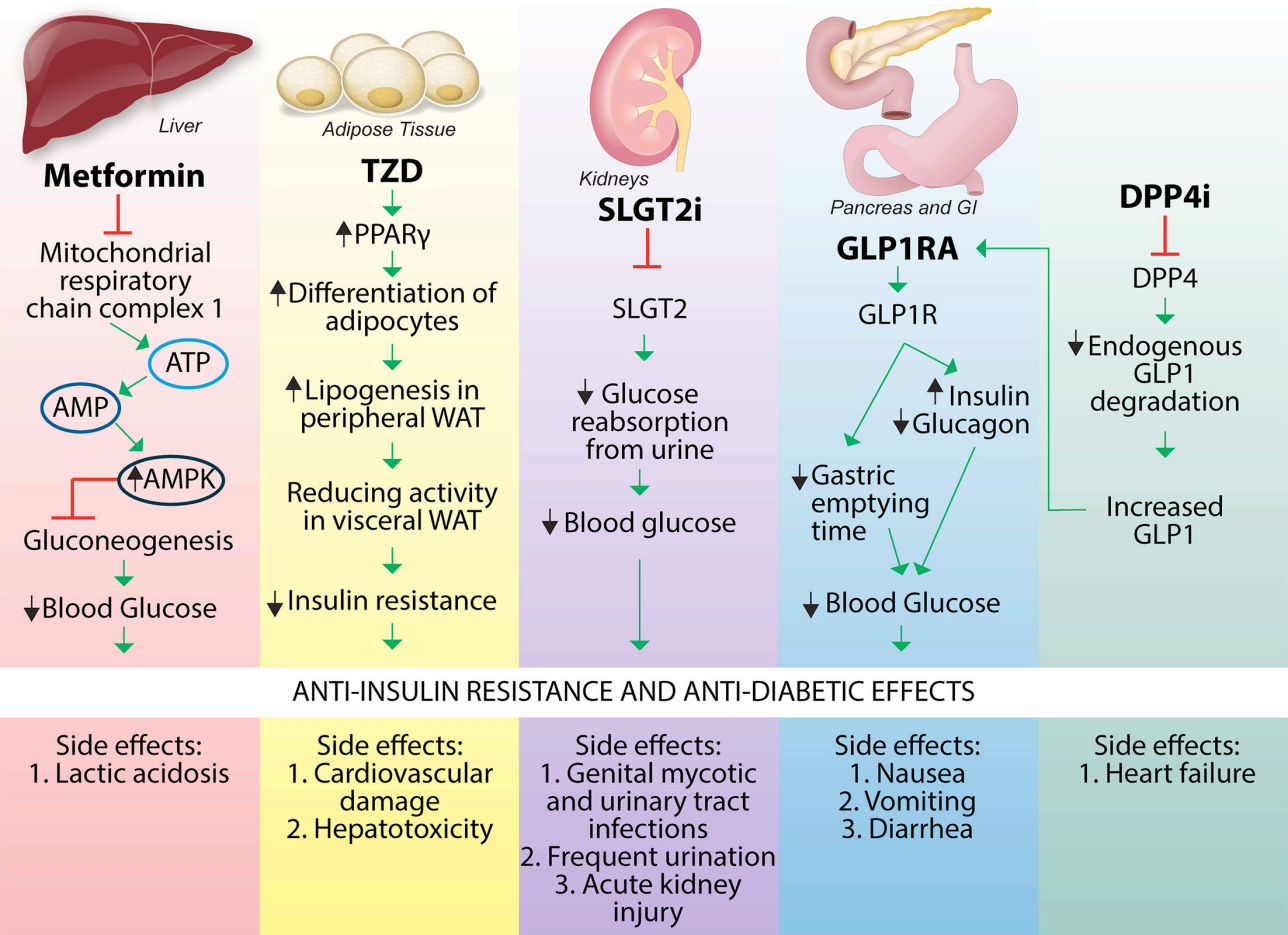
Insulin-sensitizing FDA-approved medications. The mechanism and side effects of T2DM drugs. Metformin works on hepatic mitochondria to reduce blood glucose levels. The TZDs work as PPARγ agonists in adipose tissue to reduce insulin resistance. SGLT2 inhibitors work on kidney glucose reabsorption function to lower blood glucose. GLP1 receptor agonists affect the pancreas and gastrointestinal tract to reduce blood glucose. DPP4 inhibitors increase the half-life of endogenous GLP1 to enhance GLP1 receptor function.

Some of the early insulin-sensitizing drugs that the Food and Drug Administration (FDA) approved for treating T2DM include metformin and TZDs. More recently, several compounds have been added to the arsenal against insulin resistance (Figure 9). Metformin is the most prescribed first-line antidiabetic drug, first used clinically in 1957 [154]. It was reported that metformin’s antidiabetic effect in T2DM patients was from reducing hepatic gluconeogenesis [155]. Metformin inhibits respiratory chain complex I in the mitochondria, reducing intracellular adenosine triphosphate (ATP) and increasing intracellular adenosine diphosphate (ADP) and adenosine monophosphate (AMP) levels [156]. These cause the hepatic activation of AMP-activated protein kinase (AMPK) and an AMPK-induced down-regulation of phosphoenolpyruvate carboxylase (PEPCK) and glucose-6-phosphatase (G6Pase) and the inhibition of fructose 1,6-bisphosphatase (FBP1), reducing gluconeogenesis and attenuating hyperinsulinemia [156]. Metformin-induced AMP accumulation inhibits cAMP-activated protein kinase A (PKA), a key glucagon signaling enzyme that reduces the glucose released by the liver [157]. It has been shown that metformin up-regulates HMOX1 expression in ethanol-stimulated rat intestines [158], piglet enterocytes [159], diabetic mice hearts [160], and CCl_4_-damaged rat livers [161]. Whether metformin regulates BVRA and bilirubin is not known. The mechanism of metformin’s antidiabetic effect is controversial and not completely understood. Although metformin has been popular as a first-line prescription drug for T2DM, it still causes some adverse side effects. The most reported side effect is lactic acidosis, and many T2DM patients considered at risk still receive metformin [162].

TZDs are another popular category of insulin sensitizers. They work as PPARγ agonists that promote the differentiation of adipocytes from mesenchymal stem cells, increase lipogenesis in peripheral adipose tissues, and reduce visceral adipose tissue [163], thereby inhibiting insulin resistance. PPARγ activation via TZDs induces HMOX1 expression [56,164], which could be one mechanism that improves insulin resistance. However, TZDs also cause side effects and health risks, and for these reasons, many in this class have been prohibited or highly restricted. For example, the US FDA has limited rosiglitazone use in the U.S. since 2011 because it can lead to cardiovascular dysfunction [165]. Studies show that rosiglitazone promotes cardiac hypertrophy and chromatin remodeling in isolated cardiomyocytes [166]. For these reasons, it is contraindicated in patients with congestive heart failure [167]. A study in *JAMA* concluded that people taking rosiglitazone had an increased risk of stroke, heart failure, and all-cause mortality compared with those taking pioglitazone [168]. Pioglitazone is recommended in patients with T2DM and a co-existing CVD history [169]. Troglitazone use in the United Kingdom was stopped in 2000 since it may cause hepatotoxicity [165]. Pioglitazone is still considered safe to use, but its risk of causing and worsening congestive heart failure has not been completely addressed [170].

As for alternative insulin sensitizer therapeutics, the US FDA has approved many relatively new drugs, including SGLT2i, GLP1RA, and DPP4i. SGLT2i is a glucose and sodium cotransporter expressed in proximal convoluted tubules in the kidney, which reabsorbs filtered glucose and reduces glucose excretion in urine. The SGLT2i class lowers blood glucose levels and HbA1c by inhibiting SGLT2i-dependent glucose reabsorption [171]. Some SGLT2i regulate HMOX expression and oxidative stress. For example, canagliflozin increased HMOX1 protein expression in rat vascular smooth muscle cells [172]. SGLT2i, such as empagliflozin, also reduce whole blood oxidative stress in Zucker diabetic fatty rats [173]. Dapagliflozin has been reported to suppress high-glucose-induced oxidative stress in mouse proximal tubular epithelial cells [174]. Thus, increasing HMOX1 expression and reducing oxidative stress may contribute to the SGLT2i antidiabetic effects. As for the regulation of bilirubin, a study showed that SGLT2i reduced bilirubin levels in MASLD patients [175]. There is nothing known about whether SGLT2i regulate BVR expression or activity. Studies are needed to determine whether SGLT2i affect BVR and bilirubin signaling.

The known adverse side effects of SGLT2i include genital mycotic and urinary tract infections, nausea, constipation, frequent urination [176], and acute kidney injury in patients over 65 years of age [177]. SGLT2i treatments have also been associated with an increased incidence of Fournier gangrene (FG), an uncommon but life-threatening infection-induced urologic emergency [178-180]. Although the FDA only found 19 antidiabetic drug-associated FG cases between 1984 and January 2019 [178], concerns about this have risen recently.

GLP1RA, such as semaglutide, lowers blood glucose by increasing insulin secretion, reducing glucagon secretion, and extending the gastric emptying time [181]. On the other hand, approved DPP4i, such as sitagliptin, saxagliptin, linagliptin, and alogliptin, antagonize DPP4 activity, which increases the half-life of endogenous GLP-1 and strengthens the effect of endogenous incretin hormones, resulting in reduced hyperglycemia [182].

GLP1RA modulates HMOX1 expression, bilirubin levels, and oxidative stress. For instance, liraglutide decreases blood HMOX1 and lipid hydroperoxide levels in T2DM patients [183]. However, the role of GLP1RA in regulating HMOX1 is still debated, as a study showed that a GLP1RA, exendin-4, can induce HMOX1 expression in rat renal tissues [184]. As for regulating bilirubin levels, exenatide decreased serum total bilirubin levels in T2DM patients [185]. To this end, more research is needed to determine whether GLP1RA regulates BVR. Additional studies are needed to investigate the correlations between GLP1RA and HMOX1, BVR, and bilirubin. GLP1RA may exert its antidiabetic effects by activating the GLP1 receptor, increasing HMOX1, and reducing oxidative stress.

Like GLP1RA, DPP4i’s role in regulating HMOX1 is also debated. DPP4i, such as linagliptin, do not change HMOX1 expression, as shown in a study on diabetic rats [186]. However, more studies support that DPP4i up-regulates HMOX1 expression, as saxagliptin, sitagliptin, gemigliptin, and omarigliptin have been shown to induce HMOX1 expression [187-190]. As for bilirubin regulation, sitagliptin decreased serum total bilirubin levels in rats [191], but more studies are needed to solidify the relationship between DPP4i and bilirubin. In addition, we still need evidence to confirm whether DPP4i regulates BVR and oxidative stress.

GLP1RA and DPP4i results are impactful; in some instances, people with obesity lost more than 10% of body weight [192,193]. In addition, GLP1RA is considered cardioprotective [194]. However, they still come with side effects, such as gastrointestinal and pancreatic issues, and other health risks have been found [195]. For example, GLP1RA, such as taspoglutide, lixisenatide, and exenatide, has been reported to cause nausea, vomiting, and diarrhea [195,196]. As for pancreatic issues, it has been shown that exenatide and liraglutide are associated with acute pancreatitis [197-201]. On the other hand, most of the approved DPP4i do not present cardiovascular risk [202-204], but saxagliptin is associated with a higher hospitalization rate for heart failure [205]. Although the majority support that GLP1RA and DPP4i have no adverse effects on the cardiovascular system, safety uncertainties still lead to a demand for further investigations, as we are still in the infancy of these new drugs and how they may affect the body during chronic therapies.

### Sexual dimorphism of the existing insulin resistance therapeutics

Although the prevalence of T2DM ranges from around 6% to 14% in different populations, it is similar in both sexes [4,206,207]. Interestingly, insulin resistance treatments can have sex-specific effects. For example, a combination of exenatide (a US FDA-approved GLP1RA) and metformin showed better results in reducing HbA1c in women than in men [208,209]. Liraglutide also showed better efficacy in reducing HbA1c in 18- to 64-year-old female T2DM patients than in their male counterparts [210]. Another frequently seen insulin sensitizer, rosiglitazone, has been reported to reduce HbA1c more effectively in obese female patients than in obese male T2DM patients [211]. For SGLT2i, the male sex is one of the factors increasing dapagliflozin-induced HbA1c reductions [212]. It has been reported that dulaglutide and liraglutide (GLP1RA) caused more adverse side effects in women than in men [209]. SGLT2i-related FG incidences are higher in men than women [180]. There appears to be a sexual dimorphism with these treatments, and more work is needed to understand their medical impact better.

## Sex differences in the heme oxygenase pathway and their impact on insulin resistance

### Sexual dimorphism of HMOX isotypes in insulin sensitivity

There is a sexual dimorphism in *HMOX1* gene expression that has been discussed, and it appears to play an essential role in regulating insulin sensitivity. However, whether the sexual dimorphism of HMOX2 regulates insulin sensitivity is not fully established. The effect of HMOX2 on obesity and insulin resistance has produced contrasting results in global HMOX2 knockout mice. In a study by Yao et al*.,* global deletion of HMOX2 did not affect insulin sensitivity or glucose tolerance [103]. However, HMOX2 was found to repress glycolysis and proton leak in white adipose tissue [103]. These results contrast with reports of increased obesity, hyperglycemia, and insulin resistance in global HMOX2 knockout mice [100,213]. Thus, there appear to be conflicting roles for HMOX2 in the development of obesity and insulin resistance in mice. In humans, there is limited data on the effect of the HMOX isotypes on insulin sensitivity. A study by Jais et al*.* reported that up-regulation of HMOX1 was associated with insulin resistance and inflammation in humans [101]; however, these observations have not been reproduced in subsequent studies and are not supported by the majority of preclinical studies examining the effect of HMOX1 induction on insulin resistance and inflammation. The impact of HMOX2 on insulin sensitivity in humans has yet to be extensively studied. Human polymorphisms in *HMOX2* segregate with worse markers of metabolic function in patients with polycystic ovary syndrome (PCOS) [214]. However, the effect of these polymorphisms in promoting insulin resistance in the general population has yet to be investigated.

### Induction of heme oxygenase and sex differences

The sexual dimorphism of HMOX1 inducers has not been directly tested; however, data from several animal studies suggest that sex differences in HMOX1 levels exist, potentially impacting the effectiveness of HMOX1 inducers and inhibitors between the sexes. CoPP is a potent inducer of HMOX1 that lowers fasting blood glucose and insulin while improving insulin sensitivity in both female and male mice, despite not affecting decreasing body weight in females compared with male mice [106]. CoPP treatment also increases oxygen consumption, activity, and heat production, although this effect was only studied in male mice [215]. The HMOX1 inducer, hemin, improved glucose tolerance by enhancing insulin signaling regulators such as cGMP and cAMP in the nonobese insulin-resistant type 2 diabetic Goto-Kakizaki (GK) rat [28]. Hemin treatment of Zucker diabetic-fatty rats (ZDFs) also improved insulin sensitivity through the inhibition of GSK3 and protein-tyrosine phosphatase-1B (PTP1B) and an increase in adiponectin, AMPK, and GLUT4 [104]. However, a significant limitation in both studies was the absence of females treated with hemin. Studies using adipocyte-specific HMOX1 knockout mice revealed a greater effect on body weight and composition in females than in males [216]. In addition, hyperglycemia and hyperinsulinemia were more pronounced in female adipocyte-specific HMOX1 knockout mice fed both normal and high-fat diets [216]. These results suggest that in females, adipose tissue HMOX1 may play a protective role against the development of insulin resistance compared with males.

### Bilirubin and sexual dimorphic regulation of insulin sensitivity

Similar to HMOX, there are sexual differences in plasma bilirubin levels. In 1984, Rosenthal et al. found that men of all ages have significantly higher plasma bilirubin levels than women [217]. This finding was further confirmed in a study involving over 176 million patients, which showed that males had higher bilirubin than females [218]. More studies are necessary to determine the cause of the sexual dimorphisms in bilirubin levels and whether there are differences in bilirubin signaling. Sex hormone regulation of UGT1A1 likely plays a role in these sexual differences [153], but more work is needed to fully elucidate the impact.

A potential contributor to sexual dimorphisms in bilirubin signaling is its receptor, PPARα. In rats, hepatic mRNA and protein expression of PPARα is significantly higher in males than in females, which was lost after gonadectomy, indicating the role of sex hormones [219]. The effects of sex on PPARα expression are tissue-dependent, as no differences in the heart or soleus tissue expression [219]. Female mice had 90% lower mRNA expression of PPARα in inguinal white adipose tissue (iWAT) compared with males [220]. In adipose-specific PPARα knockout mice (*Ppara* ^FATKO^), male *Ppara* ^FATKO^ mice had an increase in adiposity in the iWAT depot compared with the floxed control [220]. The increase in adiposity was not observed in female *Ppara* ^FATKO^ mice [220]. Gordon et al. demonstrated in obese male mice treated with bilirubin nanoparticles, as well as in humanized Gilbert’s syndrome mice with mild hyperbilirubinemia, that phosphorylation and activity of PPARα were significantly higher, and *Ucp1* and *Adrb3* gene expressions were substantially raised in iWAT [124]. They found that this caused a significant reduction in iWAT size and adipocyte size in the fat pad. However, their study did not include female obese mice, which is needed to elucidate the sex differences in mild hyperbilirubinemia. Future investigations are necessary to identify how sex hormones regulate PPARα and how they affect bilirubin signaling.

We have shown that urobilin is positively correlated with higher HOMA-IR in both obese men and women compared with their lean counterparts [32]. Interestingly, obese women showed three times higher urobilin levels on average than obese men [32]. Using non-targeting mass spectrometry, Ottosson et al. found that increased plasma urobilin is associated with all-cause mortality in patients with T2DM [142]. Preclinical studies have identified that urobilin is elevated in the cecal content of obese mice than in non-obese [144]. However, future investigations must be performed to determine the function of urobilin and whether it has sexual dimorphisms in its signaling.

## Brain insulin resistance and heme oxygenase, biliverdin reductase, and bilirubin

### Heme oxygenase and insulin resistance in the brain

**Figure 10:**
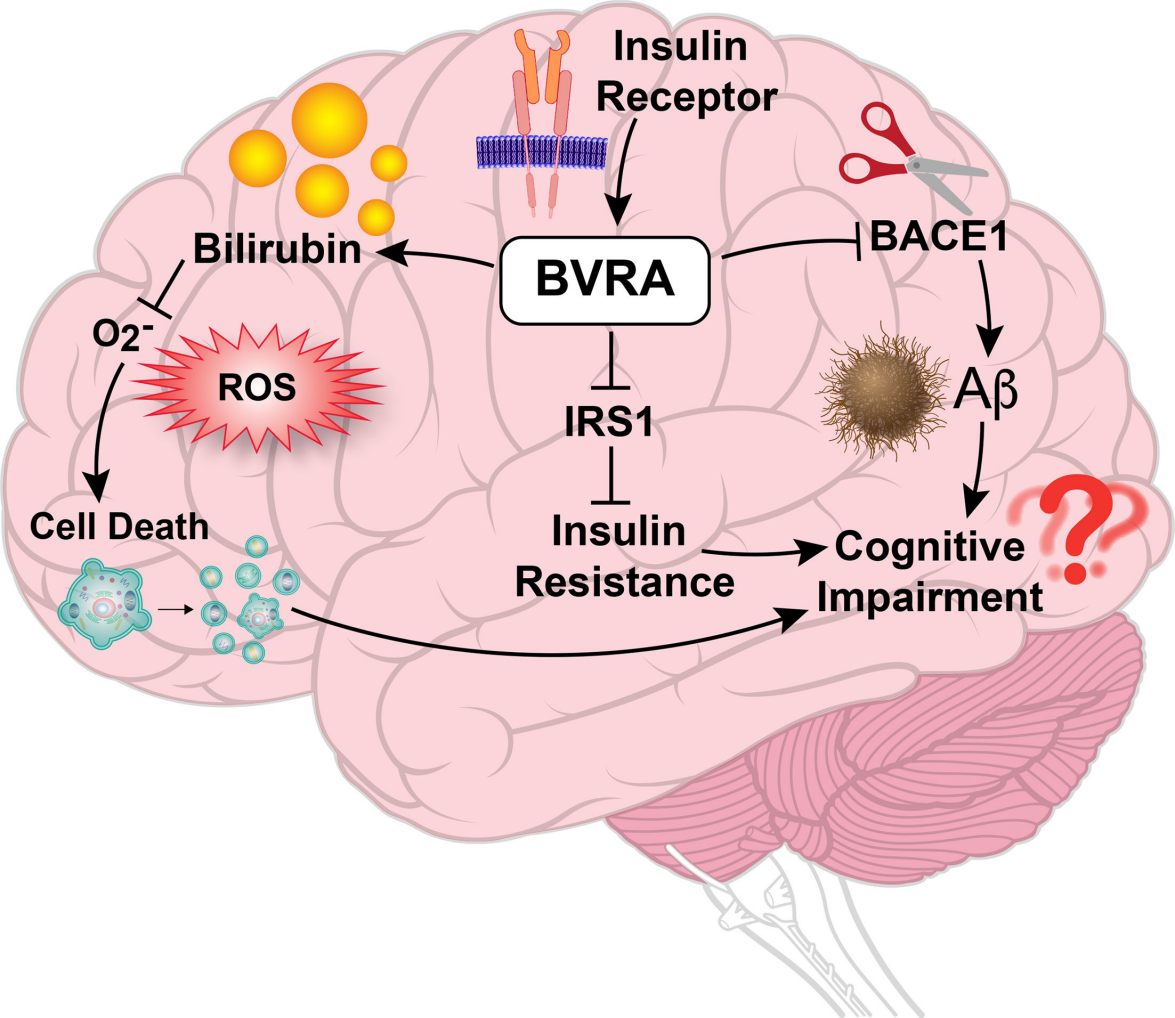
Overview of insulin receptor-induced BVRA activation and its roles in the brain and brain damage. Insulin receptors enhance the function of BVRA in the brain. BVRA helps maintain proper insulin signaling by preventing the overactivation of IRS1. Additionally, BVRA can increase bilirubin levels, which scavenge ROS and reduce cell death in the brain. BVRA also inhibits Aβ production by targeting the rate-limiting enzyme BACE1, thereby protecting against cognitive impairment.

The brain was previously thought to be non-responsive to insulin signaling because responsive tissue insulin does not stimulate the uptake and metabolism of glucose via GLUT4 activation as it does in most other insulin-sensitive tissues [221,222]. However, it is now understood that insulin has several central and peripheral actions via the brain. Centrally, insulin increases cognitive function, reduces depression, and decreases food intake [221]. Some of these functions are due to the classical insulin signaling cascade, starting with the activation of AKT and the assembly of the mTORC1 complex [222]. This signaling cascade initiates processes related to a metabolic shift from oxidative phosphorylation to glycolysis, facilitating cell growth [222]. Insulin also stimulates the assembly of the mTORC2 complex via AKT activation [222]. In the liver, mTORC2 negatively regulates FOXO1, which suppresses HMOX1. This process results in the prevention of hyperactivation-induced mitochondrial dysfunction because excessive hepatic HMOX1 levels suppress oxidative phosphorylation and fatty acid oxidation [222]. These mechanisms have not yet been confirmed in the brain but may be important in understanding the role of insulin in cognitive function. Peripherally, insulin stimulates the brain to decrease hepatic glucose production and lipolysis and to increase reproductive competence (Figure 10) [221]. These functions are impaired by insulin resistance in the brain, which is commonly observed in T2DM, obesity, and Alzheimer’s disease (AD) [222].

In the nervous system, all HMOX isoforms are present in different localizations and concentrations [223]. All HMOX isoforms produce CO as a byproduct of the reaction, which is known to activate guanylyl cyclase, cyclooxygenase, and other signaling pathways [223]. HMOX1 is nearly undetectable during homeostatic conditions in the brain [224]. More pathological roles for HMOX1 are associated with brain aging and neurodegeneration. Studies in rodents and human post-mortem brain samples from Parkinson’s patients suggest that oxidative stress-induced expression of HMOX1 is related to excessive iron deposition and mitochondrial damage [225]. However, HMOX1 levels were significantly reduced in AD patients, suggesting a disease-specific effect [225].

### Biliverdin reductase and insulin resistance in the brain

Insulin resistance can also occur in the brain, and T2DM and brain insulin resistance are associated with an increased risk for AD [226,227]. Dysfunctional BVRA kinase activity in mice is one of the earliest identifiable features of brain insulin resistance and in the progression of AD pathology [228]. BVRA is also regulated by the insulin receptor’s kinase activity, resulting in decreased IRS-1 activation, which is essential for proper insulin signaling [229]. The rate-limiting enzyme in amyloid beta (Aβ) production, B-site APP cleaving enzyme 1 (BACE1), is regulated by BVRA in beagle dogs, and the absence of BVRA activation resulted in increased Aβ production and impaired insulin signaling [230]. Therefore, dysfunctional BVRA activity created an AD-like phenotype. Furthermore, when BVRA produces bilirubin in the brain, it has a neuroprotective effect as it helps to prevent NMDAR (N-Methyl-D-Aspartate Receptor)-induced neuronal death [231,232].

### Bilirubin’s effect on brain function

Bilirubin levels are highly associated with multiple markers of brain health that impact the likelihood of developing dementia. Total bilirubin levels are inversely related to brachial-ankle pulse velocity (baPWV), which estimates arterial stiffness and predicts future cardiovascular events [233]. Furthermore, higher total bilirubin is associated with a lower risk of silent cerebral infarction (SCI) [234], which increases the risk of a multitude of cardiovascular problems, including transient ischemic attack and dementia [233]. Therefore, the risk of SCI and bilirubin levels can predict the likelihood of dementia or other brain damage.

Similarly, deep white matter lesions (DWMLs) are related to the risk of developing impaired cognitive function, stroke, and dementia [235]. DWMLs occur without noticeable symptoms and are most common in older adults. Low to intermediate serum bilirubin levels are associated with a higher incidence of DWMLs in otherwise healthy subjects than in the high bilirubin group [234]. As bilirubin decreased from high to low levels, there was almost a two-fold increase in severe DWML [234]. Therefore, the correlations of bilirubin with baPWV, SCI, and DWMLs indicate that it may be a biomarker for detecting stroke or dementia. Recognizing this initially, before damage occurs, can allow people to take preventative care, such as potentially increasing their bilirubin levels. Bilirubin also has direct correlations with AD markers. Individuals with Aβ deposits have more indirect bilirubin (IBIL), lower albumin, and a higher ratio of IBIL to albumin than controls [235]. These data suggest that too much IBIL may worsen or hasten the progression of AD. When IBIL levels were lowered via the intravenous administration of albumin, scores on AD and dementia test scales significantly improved [235]. The administration of albumin may be an effective treatment for the symptoms or progression of AD via lowering IBIL and increasing direct bilirubin (DBIL). More needs to be learned about bilirubin, its signaling mechanisms in the brain, and how it might influence neurological diseases like dementia and AD.

### Bilirubin and diabetes-related dementia

Diabetes-related dementia (DrD) is a type of dementia that occurs in people with T2DM [236]. When comparing patients with different kinds of dementia and people with T2DM, there was a stark contrast between those with AD and DrD. Those within the DrD group had significantly lower levels of antioxidants, such as bilirubin and albumin, than those in the AD group without T2DM [236]. Antioxidants like bilirubin may affect whether someone with T2DM will develop DrD. This hypothesis is supported by MASLD being a metabolic disease [7,10,15] and bilirubin being protective against it, as liver dysfunction seems connected to brain dysfunction.

Further support for this idea is seen with PPARγ agonists. PPARγ acts as a transcription factor and increases the expression of insulin-sensitizing genes [237,238]. In animal models of AD, PPARγ agonists promoted amyloid clearance, correlating with reduced amyloid plaques and decreased deficits in the mice [239]. Pioglitazone, a specific PPARγ agonist, showed promise in being protective against dementia and reached phase III clinical trials that were ultimately ended due to failing to meet the non-futility threshold [240,241]. Pioglitazone improves liver health and is a therapeutic that improves hepatic steatosis, inflammation, and ballooning grades [242]. These findings support the idea that T2DM and liver dysfunction may be related to some types of dementia, such as DrD.

DrD is not always recognized despite the evidence that T2DM is associated with an increased risk of cognitive impairment and dementia [243]. Based on the knowledge of increased cognitive impairment in post-menopausal T2DM patients, one study aimed to put all this together and examine the role between estrogens, T2DM, and cognitive impairment [244]. The main conclusion from this study was that treating T2DM mouse models with estrogen receptor (ER) agonists alleviates cognitive and memory impairments and increases activity in the hippocampus [245]. This evidence of estrogen’s role in T2DM cognitive impairment suggests that there could be a sex difference occurring, and more investigations are needed to elucidate this fully.

## Conclusions

Our understanding of the heme oxygenase system has existed for over 100 years; however, we continue to learn more yearly. An increasing volume of literature has demonstrated the important role of HMOX and bilirubin in regulating insulin sensitivity. These studies have opened an avenue for drug development, where HMOX and bilirubin may become treatment targets for insulin resistance and T2DM. Alterations in HMOX and bilirubin could explain sex-specific differences in insulin resistance, and targeting the bilirubin metabolism pathway to reduce urobilin reabsorption may better protect women from insulin resistance than men. In addition, studies to determine the function of urobilin in women versus men and how it is regulated would be beneficial. More investigations are needed about the regulatory factors that control *BLVRA* and *BLVRB* expression and the *bilR* bacterial enzyme and their functional roles in fatty liver, MASLD, insulin resistance, insulin clearance, and T2DM. While much is known about HMOX, little is known about BVR and bilR and how they may regulate insulin signaling and fat accumulation to control metabolic diseases, and more studies should reveal their importance in the future.

## Significance statement

The global epidemic of obesity has led to the manifestation of deleterious metabolic diseases accompanied by oxidative stress, adiposity, and insulin resistance. The heme oxygenase production of bilirubin is essential in regulating reactive oxygen species (ROS) that occur with metabolic-induced diseases such as T2DM. While there is literature summarizing the progress of insulin-sensitizing medications, updating the advancements in this area and how T2DM medications may regulate these pathways provides novel insights into developing improved future therapeutics.

## Data Availability

This review article contains no datasets generated or analyzed during the current study

## Abbreviations

AD: Alzheimer’s Ddisease
ADRB3: adrenergic receptor beta 3
AKT: protein kinase B
ARE: antioxidant response element
AST: aspartate aminotransferase
ATP: adenosine triphosphate
BACH1: BTB domain and CNC homolog 1
BMI: body mass index
BMSCs: bone marrow-derived mesenchymal stem cells
BVRA: biliverdin reductase-A
CK2: casein kinase-2
CO: carbon monoxide
CPT1A: carnitine palmitoyltransferase 1A
CoPP: cobalt protoporphyrin
DBIL: direct bilirubin
DIO: diet-induced obese
DPP4i: dipeptidyl peptidase 4 inhibitors
DWMLs: deep white matter lesions
DrD: Diabetes-related dementia
ER: endoplasmic reticulum
ER: estrogen receptor
ERK: extracellular signal-regulated kinases
FASN: fatty acid synthase
FDA: Food and Drug Administration
FFAs: free fatty acids
FGF21: fibroblast growth factor 21
FKBP51: FK506-binding protein-51
GLP1RA: glucagon-like peptide 1 receptor agonists
GLUT4: glucose transporter 4
GRE: glucocorticoid response element
GSK3: glycogen synthase kinase‐3
GTT: glucose tolerance test
HFD: high-fat diet
HIF-1α: hypoxia-inducible factor 1α
HMOX: heme oxygenase
H_2_O_2_: hydrogen peroxide
HRMs: heme regulatory motifs
IBIL: indirect bilirubin
IRS: insulin receptor substrate
LDL: low-density lipoprotein
MAPK: mitogen-activated protein kinases
MASH: metabolic dysfunction-associated steatohepatitis
MASLD: metabolic dysfunction-associated steatotic liver disease
MPs: metalloporphyrins
MRP2: multidrug resistance proteins 2
MRP3: multidrug resistance proteins 3
NF-κB: Nuclear Factor-κB
NHANES: National Health and Nutrition Examination Survey
NMDAR: N-Methyl-D-Aspartate Receptor
NOX: NADPH oxidase
NRF2: nuclear factor erythroid 2-related factor 2
PI3K: phosphatidylinositol 3-kinase
PPARα: peroxisome proliferator-activated receptor alpha
ROS: reactive oxygen species
RXR: retinoid X receptor
SGLT2i: sodium-glucose cotransporter 2 inhibitors
SREBP-1C: sterol regulatory element-binding protein 1
T2DM: type 2 diabetes mellitus
TZDs: thiazolidinediones
UCB: unconjugated bilirubin form
UCP1: uncoupling protein 1
UDP-GT: UDP-glucuronyltransferase
baPWV: brachial-ankle pulse velocity
mTORC1: mammalian target of rapamycin complex 1
pAKT: phospho-AKT
